# Study on Pyroelectric Harvesters with Various Geometry

**DOI:** 10.3390/s150819633

**Published:** 2015-08-11

**Authors:** An-Shen Siao, Ching-Kong Chao, Chun-Ching Hsiao

**Affiliations:** 1 Department of Mechanical Engineering, National Taiwan University of Science and Technology, No. 43, Keelung Rd., Sec. 4, Taipei 10607, Taiwan; E-Mails: qbasic147@gmail.com (A.-S.S.); ckchao@mail.ntust.edu.tw (C.-K.C.); 2 Department of Mechanical Design Engineering, National Formosa University, No. 64, Wunhua Rd., Huwei Township, Yunlin County 632, Taiwan

**Keywords:** pyroelectric cell, cyclic energy harvesting, geometry, PZT, thermal transients

## Abstract

Pyroelectric harvesters convert time-dependent temperature variations into electric current. The appropriate geometry of the pyroelectric cells, coupled with the optimal period of temperature fluctuations, is key to driving the optimal load resistance, which enhances the performance of pyroelectric harvesters. The induced charge increases when the thickness of the pyroelectric cells decreases. Moreover, the induced charge is extremely reduced for the thinner pyroelectric cell when not used for the optimal period. The maximum harvested power is achieved when a 100 μm-thick PZT (Lead zirconate titanate) cell is used to drive the optimal load resistance of about 40 MΩ. Moreover, the harvested power is greatly reduced when the working resistance diverges even slightly from the optimal load resistance. The stored voltage generated from the 75 μm-thick PZT cell is less than that from the 400 μm-thick PZT cell for a period longer than 64 s. Although the thinner PZT cell is advantageous in that it enhances the efficiency of the pyroelectric harvester, the much thinner 75 μm-thick PZT cell and the divergence from the optimal period further diminish the performance of the pyroelectric cell. Therefore, the designers of pyroelectric harvesters need to consider the coupling effect between the geometry of the pyroelectric cells and the optimal period of temperature fluctuations to drive the optimal load resistance.

## 1. Introduction

Environmental energy harvesting has become the hot topic with regard to driving low-energy consumption systems, such as wireless sensor networks. An environmentally-friendly energy source to power these nodes with a longer duration and reduced costs of maintenance is highly desirable. Large amounts of waste heat are released as a byproduct of power, refrigeration or heat pump cycles, according to the second law of thermodynamics. Thermoelectric modules are the main way for harvesting waste heat energy from temperature fluctuations. Thermoelectric generators rely mainly on the Seebeck effect to convert a steady-state temperature difference at the junction of two dissimilar metals or semiconductors into an electromagnetic force or electrical energy. Furthermore, thermoelectric elements need temperature gradients leading to heat flow in fixed temperature differences, which cannot work in the environment temperature with spatially-uniform and time-dependent temperature fluctuations within short periods [1,2]. Alternatively, pyroelectric devices directly convert time-dependent temperature fluctuations into electricity [1,2,3,4,5,6,7,8,9]. The pyroelectric effect is the change produced in the spontaneous polarization of a non-centrosymmetric dielectric material as a consequence of the change in its temperature. The change in polarization stems from the shift in the degree of non-centrosymmetry owing to thermal fluctuations corresponding to different temperatures. This change in polarization can be used to generate an electric current and, subsequently, electric power. Hence, a temperature change in time determines a correspondent variation in the induced charge. The generated current of the pyroelectric cells is based on the pyroelectric effect, which converts a temperature variation to a corresponding electrical output. The pyroelectric current is given by [3]:

I_p_ = dQ/dt = η × P × A × dT/dt (1)

where η is the absorption coefficient of radiation; A is the electrode area; dT/dt is the temperature variation rate of the pyroelectric material; Q is the induced charge; and P is the pyroelectric coefficient of the pyroelectric material given by:

P = dP_s_/dT (2)

where P_s_ is the magnitude of the electrical polarization vector. Pyroelectric cells are sandwiched between top and bottom electrodes, as flat-plate capacitors, and poled along the axis perpendicular to the plates. P_s_ is perpendicular to the capacitor surface when its magnitude is equal to the electrode charge density. Pyroelectric materials with high pyroelectric coefficients, applied in large temperature variations over time, should be considered. Moreover, increasing the electrode surface is useful for increasing the current at parity of incident thermal power density per unit area. Cuadras *et al.* [2] used pyroelectric cells based on screen-printed PZT (Lead zirconate titanate) and commercial PVDF (Polyvinylidene fluoride) films for harvesting the thermal energy supplied to low-power autonomous sensors. The pyroelectric cells in parallel association increased the generated current. The induced current from the cyclic temperature fluctuations was used to store energies of up to 0.5 mJ, enough to drive typical autonomous sensor nodes. However, the geometry of the pyroelectric cells and the frequencies or periods of temperature fluctuations necessary to optimize the performance of pyroelectric harvesters were not discussed. Hsiao *et al.* [5] used a commercial PZT pyroelectric cell with dimensions of 9 mm × 9 mm × 0.214 mm to harvest thermal energy. The optimal period was between 3.6 s and 12.2 s. The optimal frequency or work cycle is critical to enhancing the cells’ performance by controlling the heated and unheated periods, and the optimal period must be coordinated with the optimal geometry of the pyroelectric cells if the result is to be the peak performance of the pyroelectric harvesters. Zhang *et al.* [9] developed a proposal for integrating solar radiation and wind flow fluctuations to drive pyroelectric generators. A strong and a weak wind, respectively, were used to create temperature variation rates by strong forced and natural convection. However, the temperature variation rates in the pyroelectric cells were affected by the uncertainty of the wind velocity. Furthermore, the pyroelectric cells could not be periodically and consecutively heated and cooled. A PZT pyroelectric cell with a radius of 12 mm and a thickness of 0.14 mm was used for harvesting thermal energy; the optimal cycle of about 80 s was used to test the efficiency of the proposed design. However, the optimal cycle also proved to be related to the geometry of the pyroelectric cell. Sharma *et al.* [10], using finite element analysis, studied P (VDF-TrFE-CFE) polymer in conjunction with the pyroelectric effect and forced cooling to simultaneously increase energy and power density. Two approaches, linear pyroelectric harvesting and harvesting through the Ericsson cycle, were analyzed. The maximum power densities were 19.65 mW/cm^3^ and 16.35 mW/cm^3^ for the water and air cooling processes at 0.013 Hz and 0.011 Hz, respectively. The optimizing of the geometry of the polymer in order to further improve the efficiency of the pyroelectric harvester was not discussed. However, in the design of pyroelectric harvesters, the work cycles or periods need to fit with the geometry of the pyroelectric cells.

Pyroelectric harvesters mainly use cyclic heating and cooling with a periodic temperature profile to convert temperature fluctuations into electricity. Although the frequency or work cycle in the periodic temperature profile is a critical factor for improving efficiency, the geometry of pyroelectric cells is also important as it depends on the optimal period. This research proposes a complete analysis involving simulated calculations and experiments for revealing the relationships between the working frequency in the periodic temperature profile and the geometry of pyroelectric cells under constrained material properties of pyroelectric cells.

## 2. Materials and Methods

### 2.1. Fabrication of Pyroelectric Cells

**Figure 1:**
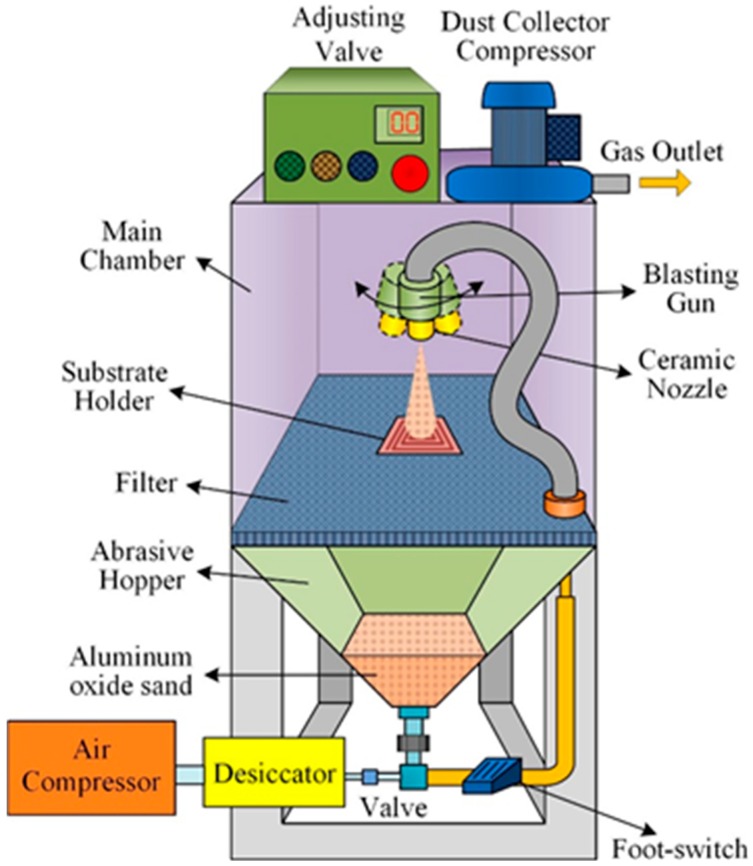
Schematic diagram of the sandblast etching machine.

A PZT pyroelectric cell with the dimensions of 9 mm × 9 mm × 0.414 mm was used. The cell comprised a 0.4 mm-thick PZT sheet sandwiched between a top and a bottom electrode. The electrodes were of 7 μm-thick silver film. The PZT cells were provided by Eleceram Technology Co. Table 1 shows the properties of the commercial PZT pyroelectric cell. Hsiao *et al.* [4] used a sandblast etching technique to trench complex patterns in commercial PZT sheets to improve the temperature variation rate and enhance the efficiency of pyroelectric harvesters. Thus, sandblast etching has been proven to be a beneficial technique for fabricating various geometries and thicknesses of pyroelectric cells. The schematic diagram of the sandblast etching apparatus is depicted in Figure 1. This machine, molded into a dry blasting machine, was manufactured and assembled by the Shang-Po Sander Co. Ltd. (New Taipei city, Taiwan). A dry sandblasting machine generally consists of six systems: (i) the structural system; (ii) the media power system; (iii) the piping system; (iv) the dust removal system; (v) the control system; and (vi) the auxiliary system. The working principle of sandblast etching is that compressed air carrying aluminum oxide sand at high speed collides with the surface of the workpiece; compressed air flows through a valve to form negative pressure and the aluminum oxide sand is drawn into the blasting gun. Aluminum oxide sand is accelerated and spurted from the ceramic nozzle to etch the substrates.

**Table 1:** Properties of the commercial PZT pyroelectric sheet.

Sample ID	Thickness (μm)	Area (mm^2^)	Size (mm × mm)	Relative Dielectric Constant (ε_33_^T^/ε_O_)	Density (g/cm^3^)	Poling Field (V/μm)	Pyroelectric Coefficient (10^−4^·C·m^−2^·K^−1^)
KA	400	81	9 × 9	2100	7.9	3.5	6.97

**Figure 2:**
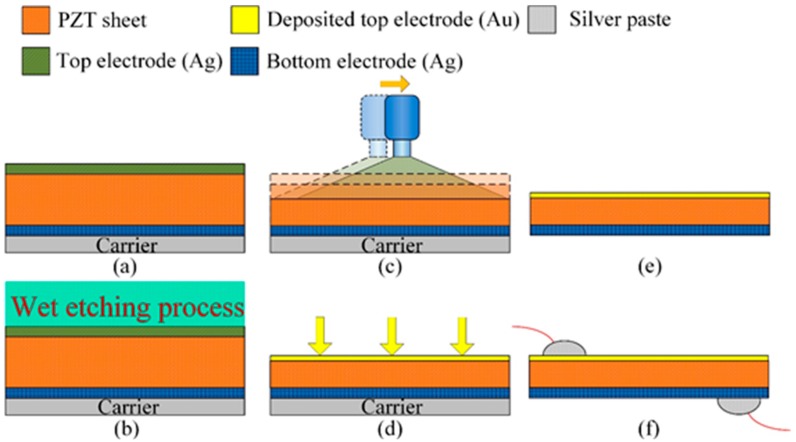
Fabrication flow of the PZT pyroelectric cell.

**Figure 3:**
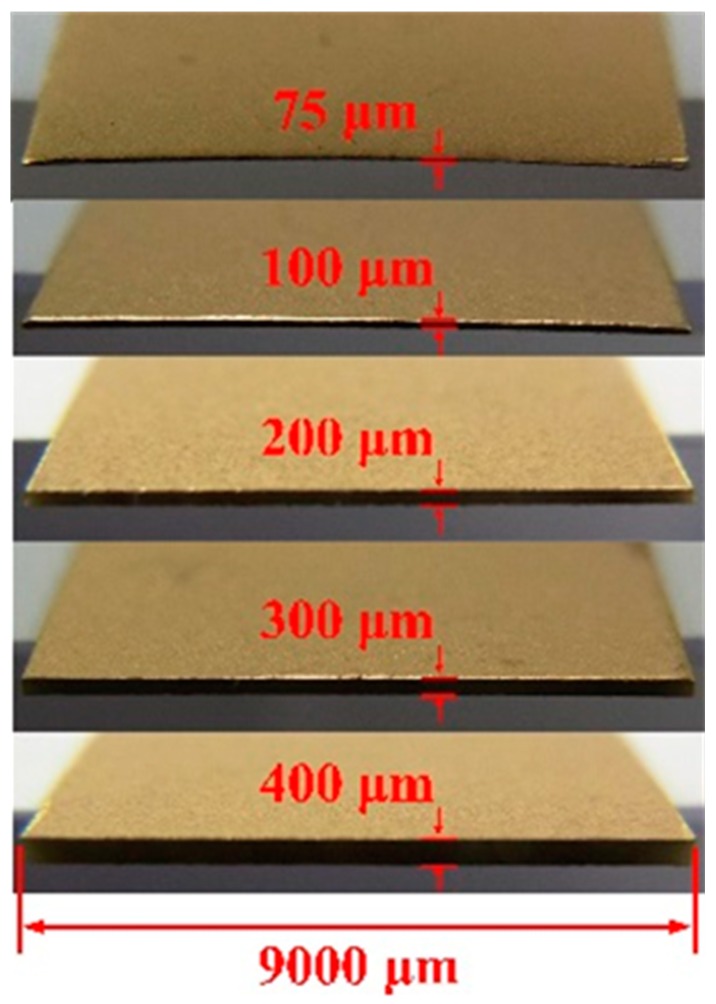
Fabricated pyroelectric cells with various thicknesses.

The process flow of the PZT pyroelectric cells is shown in Figure 2. First, the PZT sheet was attached to a carrier of glass substrate, as shown in Figure 2a. Wet etchant of HNO_3_:H_2_O = 7:3 was used to remove the top electrode, as shown in Figure 2b. The sandblast etching machine was used to manufacture PZT cells with various thicknesses, as shown in Figure 2c. Then, a 100 nm-thick gold film was deposited on the top side of the PZT sheets to produce the top electrode using an E-beam evaporator, as shown in Figure 2d. Finally, the carrier was removed to achieve the fabrication of the PZT pyroelectric cells, as shown in Figure 2e. The top and the bottom electrodes were connected with copper wires by a silver paste for measuring the electrical properties, as shown in Figure 2f. The fabricated pyroelectric cells with various thicknesses are shown in Figure 3.

### 2.2. Thermodynamic and Electric Models

**Figure 4:**
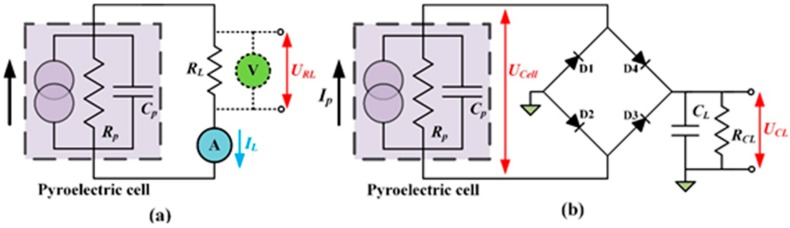
Model of electric energy harvesting process using pyroelectric cells: (**a**) directly driving a load resistance (*R_L_*); (**b**) using a bridge rectifier to store the electrical energy in a storage capacitor (*C_L_*).

Pyroelectricity is an electrical current generation from time-dependent temperature fluctuations. The pyroelectric cell is used to convert temperature fluctuations into the induced charge, which is usually considered to be a current source in parallel with its equivalent capacitance, *C_P_*, and resistance, *R_P_*. The thermodynamic governing equations were constructed according to a lumped parameter thermodynamic model, which was adopted to estimate the transient thermal response of the pyroelectric cell, as follows [9]:

(3) $$\{\begin{array}{c} \begin{array}{c} {\alpha Q}_{in}=C_{T}\frac{dT\;\left(t\;\right)}{dt}+\frac{T\;\left(t\;\right)\;{-\;T}_{air}}{R_{conv}}{+Q}_{loss}{+Q}_{cell}\\ Q_{loss}\;=\;\varepsilon \;A\;\sigma \;\left(T{\left(t\right)}^{4}{-T}_{air}^{\;4}\right)\\ Q_{cell}{=I}_{p}\left(t\right)\;U_{cell}\left(t\right) \end{array}\\ R_{conv}=1/\left(h\times 2A\right)\\ C_{T}{=\rho \;C}_{cell}{\;L}^{\;2}\;d \end{array}$$

where *Q_in_* is the input radiation power; *Q_loss_* is the heat loss from the cell to the environment; *Q_cell_* is the power generation of the cell; α is the heat absorption rate of the pyroelectric material; *T(t)* is the transient temperature in the cell; *T_air_* is the ambient temperature; *C_T_* is the thermal capacitance of the cell; *C_cell_* is the specific heat of the cell; A is the electrode area of the cell; *h* is the natural convection coefficient of the cell; *R_conv_* is the thermal resistance caused by convection; *I_p_* is the pyroelectric current; *U_cell_* is the generated voltage in the cell; σ is the Stefan-Boltzmann constant (5.67 × 10^−8^ W·m^−2^·K^−4^); ε is the emissivity on the irradiated surface of the cell; ρ is the density of the cell; *L* and *d* are the width and thickness, respectively, of the cell with a square sheet. Furthermore, the current *I_p_* can be determined using Equation (1). The circuit analysis was divided into two parts: first, driving a load resistance (*R_L_*); and second, using a bridge rectifier to store the electrical energy in a stored capacitor (*C_L_*), as depicted in Figure 4.

According to Kirchhoff's current law, at any node (junction) in an electrical circuit, the sum of the currents flowing into that node is equal to the sum of the currents flowing out of that node. *U_RL_* is the voltage in the load resistance. The *I_L_* is the current through the load resistance, which can be expressed as follows:

(4) $$\{\begin{array}{c} I_{p}\left(t\right){=C}_{p}\frac{{dU}_{RL}\left(t\right)}{dt}+\frac{U_{RL}\left(t\right)}{R_{p}}+\frac{U_{RL}\left(t\right)}{R_{L}}\;\\ I_{L}\left(t\right)=\frac{U_{RL}\left(t\right)}{R_{L}}\; \end{array}$$

Considering the stored energy, a full-wave rectifier circuit was used to store the induced charge generated from thermal oscillations in a storage capacitor, *C_L_*. *C_L_* was a 4.7 μF electrolytic capacitor with a 50 V maximum voltage. During the heating cycle, the two forward-biased diodes (D1 and D3) allowed the generated current flow, *I_p_*, through to charge *C_L_*. The other diodes (D2 and D4) were reverse-biased and blocked the current flow. During the cooling cycle, the direction of *I_p_* was reversed, and *C_L_* was charged through D2 and D4. The measured forward voltage drop (*V_d_*) of the diodes (Model 1N4148, IHS, Englewood, CO, USA) was 0.62 V. *U_CL_* is the stored voltage in the storage capacitor, *C_L_.* *R_CL_* is the electric loss. The governing equation for the electric model can be expressed as follows:

(5) $$\{\begin{array}{cc} \begin{array}{c} I_{p}\left(t\right){=C}_{p}\frac{{dU}_{cell}\left(t\right)}{dt}+\frac{U_{cell}\left(t\right)}{R_{p}}\\ C_{L}\frac{{dU}_{CL}(t)}{dt}=\frac{U_{CL}\left(t\right)}{R_{L}} \end{array} & \left(|U_{cell}|≦\;|U_{CL}|+\;2\;V_{d}\right) \end{array}$$

(6) $$\{\begin{array}{cc} \begin{array}{c} I_{p}\left(t\right){\mathrm{=C}}_{p}\frac{{dU}_{cell}\left(t\right)}{dt}+\frac{U_{cell}\left(t\right)}{R_{p}}{+C}_{L}\frac{{dU}_{CL}(t)}{dt}+\frac{U_{CL}(t)}{R_{CL}}\\ {|U}_{cell}{|=|U}_{CL}{|+2\;V}_{d}\; \end{array} & \left(|U_{cell}|>\;|U_{CL}|+\;2\;V_{d}\right)\; \end{array}$$

When the generated voltage of the pyroelectric cell is lower than the sum of the stored voltage and double forward voltage, the bridge rectifier is useless and the cell is considered electrically-insulated from the storage capacitor, as shown in Equation (5). Nevertheless, when the generated voltage of the pyroelectric cell is higher than the sum of the stored voltage and double forward voltage, the bridge rectifier is useful, and the storage capacitor is charged from the pyroelectric cell with thermal fluctuations via the bridge rectifier, as shown in Equation (6).

**Figure 5:**
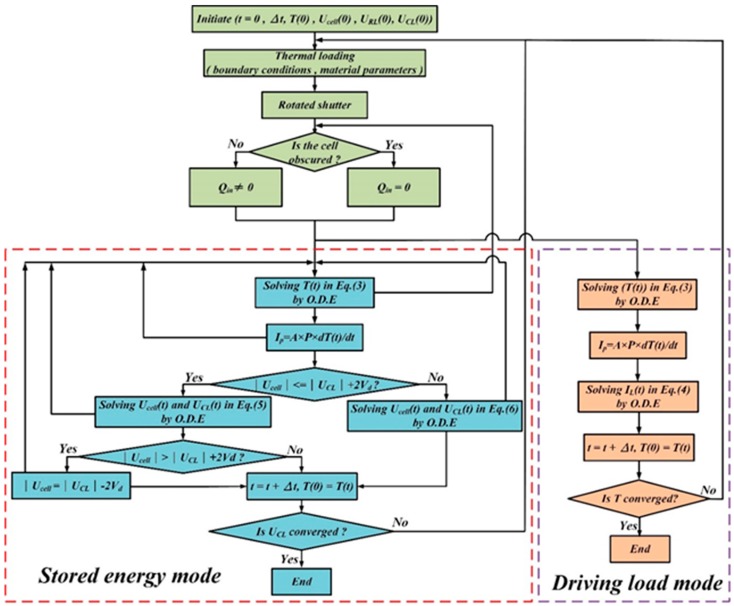
Theoretical analysis for integrating the thermodynamic and the electric models of pyroelectric cells.

Figure 5 shows a numerical calculation flow for combining the thermodynamic and electric models of the pyroelectric cell. The time step Δ*t* is related to the accuracy of the numerical analysis. The initial temperature *T(t = 0)* is set as ambient temperature *T_air_*. The initial voltages in the pyroelectric cell and the storage capacitor are *U_cell_(t = 0) = 0* and *U_CL_(t = 0) = 0*, respectively. The pyroelectric cell heated when the shutter did not cover the cell and *Q_in_* was used for the numerical calculation, and the pyroelectric cell cooled when the shutter covered the cell and *Q_in_* was set to zero. The thermal energy harvesting process was, first, to calculate the transient temperature variation *T(t)* by using the ordinary differential equations (ODE) solver in the commercial software MATLAB R2014a. The generated pyroelectric current *I_p_* was determined from the difference between the initial and last temperature values in this step. The electric model followed the thermodynamic model. The state of the bridge rectifier was first determined, then the charge flow needed to maintain the newly induced charge on the cell or to deliver the induced charge to be stored in the storage capacitor. Moreover, *C_p_* was much smaller than *C_L_*. *T(t)*, *U_cell_(t)*, *U_RL_(t)*, and *U_CL_(t)* were updated by the last calculated value by means of the computations of the ODE solver. The relative conditions for the theoretical analysis are shown in Table 2.

**Table 2:** Relative parameters for the theoretical analysis.

A	h	*P*	*T_air_*	*V_d_*	α	ε	σ	*Q_in_*	*C_cell_*
81 × 10^−6^ (m^2^)	20 (W·m^−2^·K^-1^)	580 (μC·m^−2^·K^−1^)	297 (K)	0.62 (V)	0.5	0.5	5.67 × 10^−8^ (W·m^−2^·K^−4^)	2500 (W)	360 (J·kg^−1^·K^−1^)

### 2.3. Measurement

**Figure 6:**
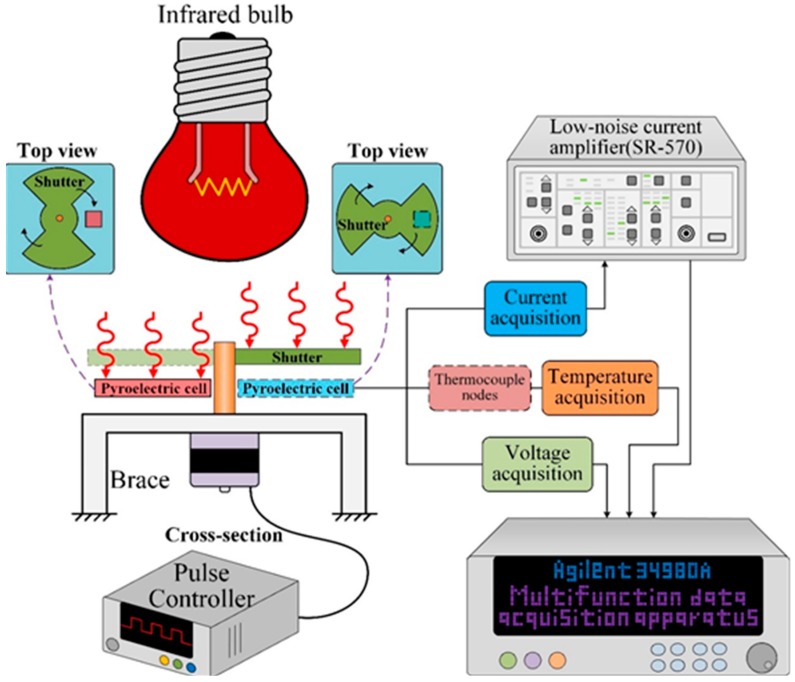
Measurement setup for the pyroelectric harvester.

Pyroelectric harvesters mainly use a periodic temperature profile for generating temperature fluctuations in pyroelectric cells. Figure 6 shows a schematic diagram of the pyroelectric harvester for harvesting cyclic heat energy and integrating electrical with thermal systems to frame a measurement setup. The measurement setup consisted of a shutter, a gear motor, an infrared bulb (Heat Plus 250 W, 110 V, Philips, Amsterdam, Netherlands), thermocouple sensors, and the PZT pyroelectric cells placed on a printed circuit board (PCB). Type K (Chromel/Alumel) thermocouple sensors were used to measure temperature variations in the PZT pyroelectric cell; they were attached to the bottom electrode at the center of the PZT cell, thereby ensuring good thermal contact. The distance between the infrared bulb and the PZT cell was about 6.5 cm. The gear motor was controlled at various rotary speeds to drive the shutter and cause further temperature oscillations in the cell via a pulse controller. The infrared bulb with the shutter resulted in temperature fluctuations in the range of 70 °C to 80 °C. The shutter was divided into four parts, and two heating and cooling zones were generated as the shutter rotated through the cycle. The infrared lamp was the heat source, which was projected onto the PZT pyroelectric cells through the gaps in the shutter. In this way, the shutter prevented the PZT cells absorbing heat from the heat source except through the gaps in the shutter. When the shutter rotated, the PZT pyroelectric cell was periodically heated and cooled. Finally, the output data of temperature, current and voltage were measured simultaneously with a computer-controlled data acquisition apparatus (34980A, Agilent Technologies Co. Ltd., Santa Clara, CA, USA), and a low-noise current amplifier (SR-570, Stanford Research Systems Inc., Santa Clara, CA, USA) was used to carefully confirm the current generated from the pyroelectric harvester and remove interferences.

## 3. Results and Discussion

Using the pyroelectric effect is an innovative cyclic heating scheme whereby thermal energy can be converted into electricity. A periodic temperature profile generated by a cyclic heating system can be applied to the pyroelectric harvester. In other words, the temperature of the pyroelectric materials varies between hot and cold regions with various frequencies or periods. Furthermore, the optimal period of temperature fluctuations is directly related to the geometry of the pyroelectric cells.

**Figure 7:**
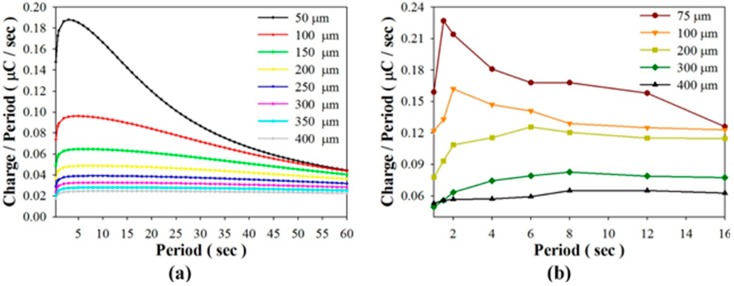
Relationships between the induced charge rate and various periods when using PZT cells of various thicknesses, as determined by simulated calculation (**a**); and experiment (**b**).

In the open circuit mode, Figure 7a shows the relationships between the induced charge rate and various periods when using PZT cells with thicknesses of 50, 100, 150, 200, 250, 300, 350, and 400 μm. It was obvious that the induced charge rate was directly related to the period in which the thinner PZT cell was adopted. Moreover, the optimal period increased when the thickness increased; thus, the thinner the PZT cell, the larger the induced charge rate. The induced charge rate showed a rapid increase for thicknesses under 150 μm. Therefore, enhancing the efficiency of the pyroelectric harvester clearly depended on the thickness of the pyroelectric cells and the work period. An experiment was conducted to prove these results. As depicted in Figure 7b, the optimal periods were about 1.5 s, 2 s, 6 s, 8 s, and 12 s when using PZT cells with thicknesses of 75 μm, 100 μm, 200 μm, 300 μm, and 400 μm. Furthermore, the induced charge rate of the 75 μm-thick PZT cell was extremely dependent on the period. The performance of the pyroelectric harvester with the thinner pyroelectric cell immediately declined when the period was not optimal.

**Figure 8:**
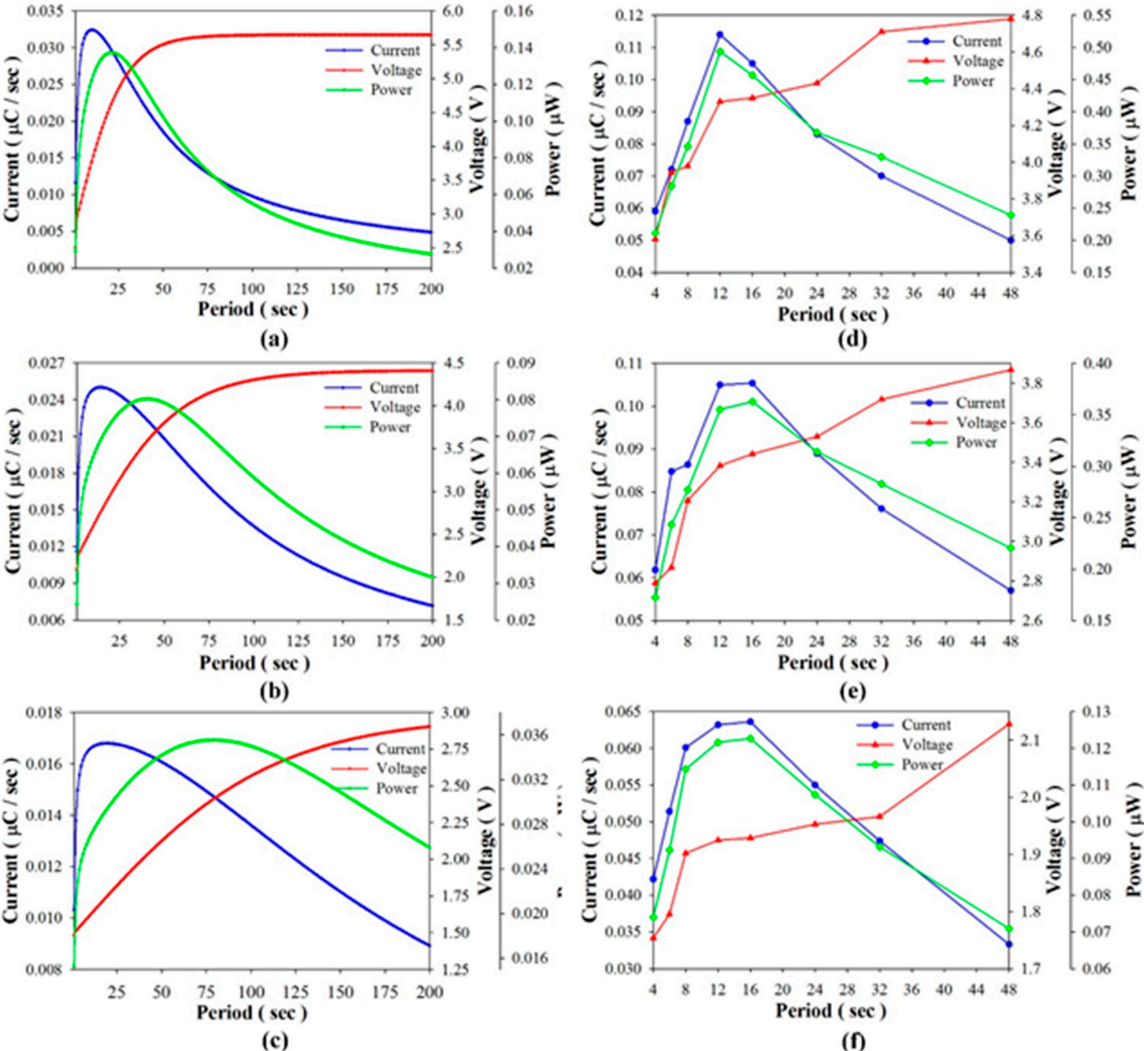
Harvested power, voltage and current as functions of the period when using PZT cells with thicknesses of 100 μm (**a**,**d**), 200 μm (**b**,**e**), and 400 μm (**c**,**f**) to directly drive a 44 MΩ load resistance as determined by the simulated calculation (**a**–**c**) and the experiment (**d**–**f**).

**Figure 9:**
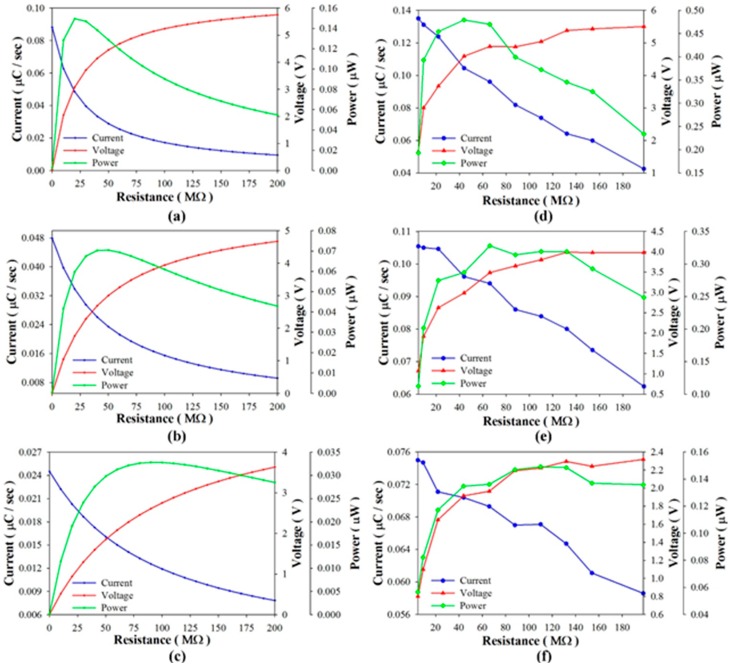
Harvested power, voltage and current as functions of the load resistance when using PZT cells with thicknesses of 100 μm (**a**,**d**), 200 μm (**b**,**e**), and 400 μm (**c**,**f**) at a constant period of 16 s as determined in the simulated calculation (**a**–**c**) and the experiment (**d**–**f**).

**Figure 10:**
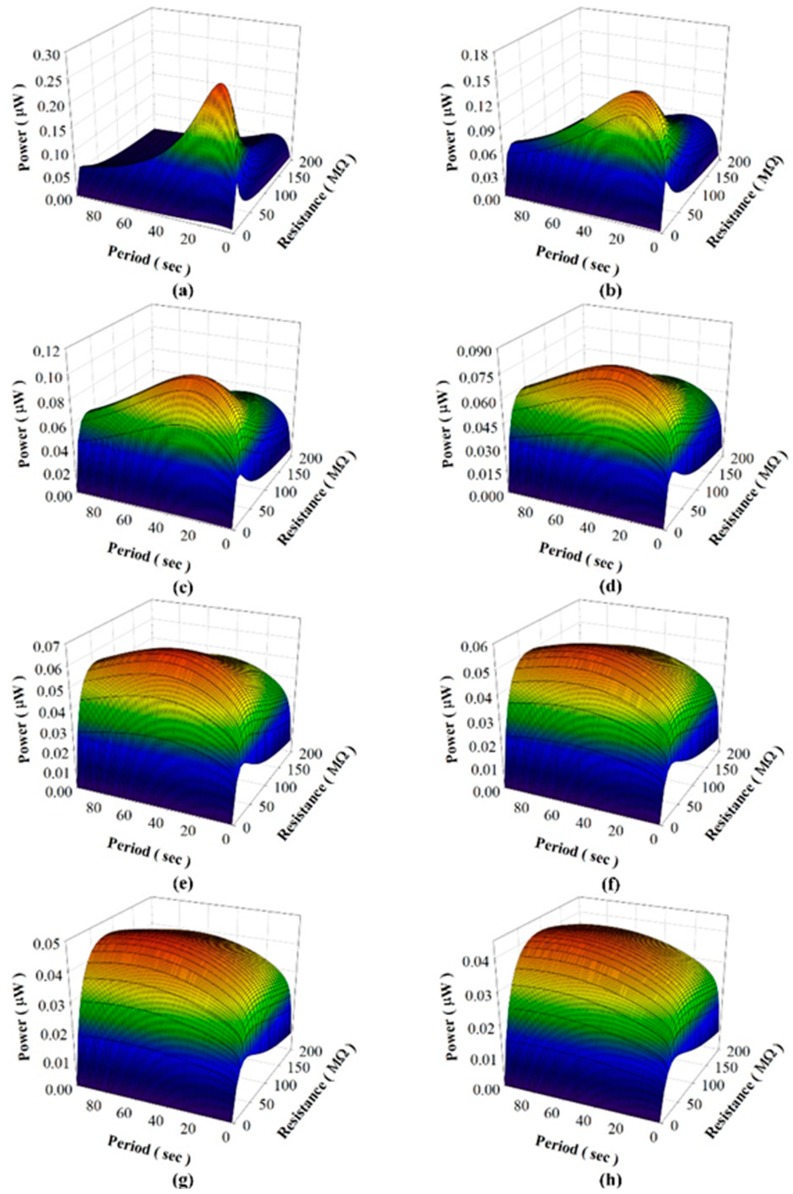
Relationships between the harvested power, load resistance and period when using PZT cells with thicknesses of 50 μm (**a**); 100 μm (**b**); 150 μm (**c**); 200 μm (**d**); 250 μm (**e**); 300 μm (**f**); 350 μm (**g**); and 400 μm (**h**).

Considering that the pyroelectric harvester directly drives load resistances, Figure 8 shows the harvested power, voltage, and current as functions of the period when using PZT cells of various thicknesses to drive a 44 MΩ load resistance in the simulated calculation and the experiment. The experimental data showed that the optimal period was about 12 s when using the 100 μm-thick PZT cell, and in a range of 12 s~18 s when using the 400 μm-thick PZT cell. The harvested power of the thinner PZT cell was higher than that of the thicker PZT cell for the optimal period. However, the efficiency of the pyroelectric harvester with the thinner PZT cell was greatly reduced when the working period diverged even slightly from the optimal period. Moreover, the tendency of the harvested power curves in the simulation was similar to that in the experiment. Figure 9 shows the harvested power, voltage and current as functions of the load resistance when using PZT cells of various thicknesses at a constant period of 16 s in the simulated calculation and the experiment. The harvested power was at the maximum when the 100 μm-thick PZT cell was used to drive the optimal load resistance of about 40 MΩ. Again, the harvested power was greatly reduced when the working resistance diverged slightly from the optimal load resistance. The efficiency of the pyroelectric harvester was greatly dependent on the load resistance when using the thinner PZT cell. This tendency was further evident in the relationships between the harvested power, load resistance and period as determined by the simulated calculation, as shown in Figure 10. Hence, the use of the thinner PZT cell required the simultaneous matching of the optimal period with the load resistance in order to improve the efficiency of the pyroelectric harvester. Although the use of the thinner PZT cell could increase the performance of the harvester, a mismatched combination of period and load resistance resulted in a serious reduction in harvested power and negated the benefit gained in using the thinner rather than the thicker PZT cell.

**Figure 11:**
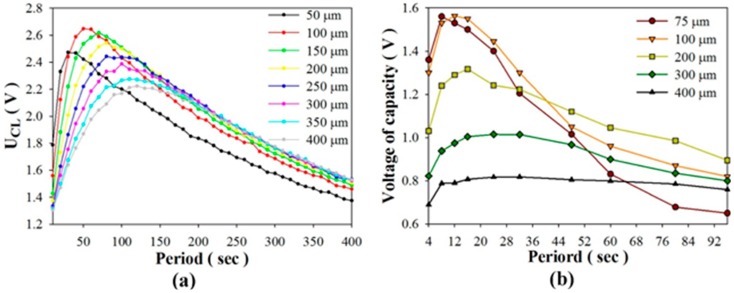
Relationships between the stored voltage and various periods when using PZT cells with various thicknesses as determined in the simulated calculation (**a**); and the experiment (**b**).

**Figure 12:**
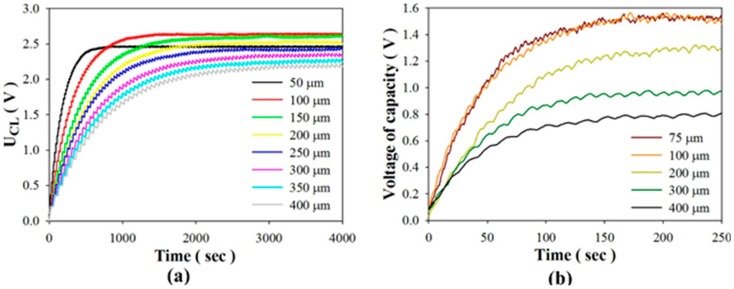
Relationships between the stored voltage and time when using PZT cells with various thicknesses, respectively, at the optimal period, as determined in the simulated calculation (**a**); and the experiment (**b**).

As to the stored energy mode, Figure 11 shows the relationships between the stored voltage and various periods when using PZT cells of various thicknesses. Stored voltage is defined as a stable voltage when the induced charge generated from a PZT pyroelectric cell with temperature fluctuations is used to store the electrical energy in a 4.7 μF electrolytic capacitor. The experimental data clearly showed that the performance of the PZT cell was greatly dependent on the period of use of the thinner PZT cell. The optimal periods to maximize the stored voltage were located at about 8 s, 12 s, 16 s, 24 s and 32 s when using PZT cells with thicknesses of 75 μm, 100 μm, 200 μm, 300 μm, and 400 μm, respectively. The thinner PZT cell possessed the shorter optimal period. The stored voltage generated from the 100 μm-thick PZT cell was less than that from the 200 μm-thick PZT cell for a period longer than 40 s. Furthermore, the stored voltage generated from the 75 μm-thick PZT cell was less than that from the 400 μm-thick PZT cell for a period longer than 64 s. Moreover, the maximum stored voltage of the 75 μm-thick PZT cell was about 8% lower than that of the 100 μm-thick PZT cell. Although the thinner PZT cell could improve the efficiency of the cell, any divergence in the optimal period would immediately reduce its performance. Figure 12 shows the relationships between the stored voltage and time when using PZT cells of various thicknesses for the optimal period. Although the thinner PZT cell could enhance the stored voltage, the much thinner 75 μm-thick PZT cell was disappointing due to the thinner PZT pyroelectric cell with a larger capacitance to decrease the induced voltage. The decrease in the induced voltage reduced the voltage difference between the PZT cell and the storage capacitor, which caused a decrease in the charge flow to the storage capacitor. Therefore, hoped-for improvements to the efficiency of pyroelectric harvesters require a thorough exploration of the relationships between the thickness of the pyroelectric cells and the working period under constrained material properties of pyroelectric cells.

## 4. Conclusions

This aim of this study was, by means of simulated calculations and experiments, to determine the relationships between the geometry of pyroelectric cells and the optimal period of temperature oscillations driving the optimal load resistance in order to further improve the efficiency of pyroelectric harvesters. Although the induced charge generated from the thinner PZT cell was increased, it was rapidly reduced when the period diverged from the optimal. The harvested power was at the maximum when the 100 μm-thick PZT cell was used to drive the optimal load resistance of about 40 MΩ, but it was greatly reduced under a working resistance which even slightly diverged from the optimal load resistance. Moreover, the stored voltage generated from the 75 μm-thick PZT cell was less than that from the 400 μm-thick PZT cell for a period longer than 64 s. Although the thinner PZT cell could enhance the performance of the pyroelectric harvester, the much thinner 75 μm-thick PZT cell and any divergence in the period disappointingly resulted in the reduced efficiency of the cells. Therefore, the designing of pyroelectric harvesters must include considerations as to the coupling effect between the geometry of the pyroelectric cells and the optimal period of temperature oscillations to drive the optimal load resistance as per the proposed analysis method.
